# Epigenetic and Transcriptomic Impacts of Ethanol Vary by Brain Region and Extent of Exposure

**DOI:** 10.1523/ENEURO.0484-25.2026

**Published:** 2026-04-09

**Authors:** Erica M. Periandri, Kala M. Dodson, Mariana Lopes, Francisca N. de Luna Vitorino, Anjola Ola, Joanna M. Gongora, Benjamin A. Garcia, Karl M. Glastad, Gabor Egervari

**Affiliations:** ^1^Department of Genetics, Washington University School of Medicine, St. Louis, Missouri 63110; ^2^Department of Nutrition, Case Western Reserve University School of Medicine, Cleveland, Ohio 44106; ^3^Department of Biochemistry and Molecular Biophysics, Washington University School of Medicine, St. Louis, Missouri 63110; ^4^Department of Biology, University of Rochester, Rochester, New York 14627

**Keywords:** acute alcohol exposure, brain, chronic alcohol exposure, epigenetics, ethanol, transcriptomics

## Abstract

Epigenetic and transcriptional mechanisms are key contributors to alcohol use disorder (AUD). However, a better understanding of the specific genes, transcripts, and chromatin marks affected is necessary to inform novel pharmacotherapies. Here, we systematically investigate the genome-wide epigenetic and transcriptomic effects of ethanol across key brain regions relevant to AUD and assess how these outcomes differ between acute and chronic exposure in male C57BL/6J mice. We show that alcohol-derived acetate contributes to histone acetylation in the brain in response to acute or chronic exposure, with a broader and more robust effect following repeated exposure. Further, we find that chromatin and transcriptomic changes elicited by acute or chronic ethanol exposure are predominantly specific to brain region and observe more robust dysregulation of gene and transcript expression following acute exposure. We show that ethanol-induced transcriptional changes are paradigm dependent in some brain regions, most strikingly in the ventral hippocampus. Overall, our results systematically illuminate and compare key epigenetic and transcriptomic outcomes linked to acute and chronic ethanol exposure, which will guide the development of future therapeutic interventions.

## Significance Statement

This is the first study to systematically investigate epigenetic and transcriptomic changes following acute or chronic exposure to alcohol, focusing on key regions previously linked to substance use disorders. We show the molecular impact of alcohol varies among brain regions and in part depends on the extent of alcohol exposure. Our results provide unprecedented detail on how alcohol affects transcriptional regulation in the brain, which in turn will inform the development of needed novel therapeutic interventions for alcohol use disorder.

## Introduction

Despite decades of research on its detrimental consequences, the use and misuse of alcohol continues to be widespread. Alcohol is the most commonly used psychoactive substance of significant public health importance (World Health Organization, 2024), and alcohol use disorder (AUD) is among the most prevalent substance use disorders in the United States (Substance Abuse and Mental Health Services Administration, 2025). However, pharmacotherapies for AUD are limited and lack sustained efficacy, largely due to the complex and widespread molecular effects of alcohol on the brain (Ron and Barak, 2016; Egervari et al., 2021).

Postmortem and translational studies consistently link chronic alcohol consumption with epigenetic (Zakhari, 2013; Finegersh et al., 2015; Berkel and Pandey, 2017; Mahnke et al., 2017; Palmisano and Pandey, 2017) and transcriptomic changes including alternative splicing (Van Booven et al., 2021; Huggett et al., 2023; Carvalho and Lasek, 2024; Bhatnagar and Heller, 2025) in the brain. However, the mechanisms contributing to these molecular signatures remain ambiguous. Further, there is a current lack of knowledge on how ethanol-induced epigenetic and transcriptomic outcomes differ between acute and chronic exposure. A better understanding of these differences can provide novel insight into mechanisms that play a key role during the transition from limited to chronic consumption.

Recent findings outlined a metabolic-epigenetic nexus by which acetate produced from ethanol metabolism directly contributes to histone acetylation in the mouse hippocampus, regulating alcohol-associated learning (Mews et al., 2019). This incorporation is mediated by Acetyl-CoA Synthetase 2 (ACSS2), a metabolic enzyme that converts acetate into acetyl-CoA, which is the primary substrate of histone acetyltransferases (HATs) and is necessary for histone acetylation (Mews et al., 2017). Follow-up work addressed the relevance of ACSS2 in a model of voluntary binge alcohol drinking, “Drinking in the Dark” (DID; Egervari et al., 2025). Interestingly, ACSS2 was found to regulate voluntary ethanol consumption, primarily in male mice: Knock-out of ACSS2 resulted in decreased alcohol drinking and blunted alcohol-related gene expression and histone acetylation changes in the ventral striatum (VS) of male mice following DID (Egervari et al., 2025).

It remains unknown whether ethanol-derived acetate directly contributes to histone acetylation in the VS and other nonhippocampal brain regions implicated in maladaptive behaviors associated with AUD. Further, it has not been investigated how this incorporation may differ between acute and chronic exposure to alcohol. Overall, the epigenetic and transcriptomic effects of acute or chronic ethanol exposure are yet to be systematically assessed across key brain regions relevant to AUD.

Here, we use a combination of advanced proteomic and transcriptomic approaches to query the epigenetic and transcriptomic impacts of acute and chronic alcohol exposure across the mouse brain, focusing on key regions associated with AUD: the cortex, hippocampus, ventral striatum, and dorsal striatum (Koob and Volkow, 2016). We show alcohol-derived acetate is incorporated into histone acetylation in the brain in response to acute or chronic exposure, with a broader and more robust effect following repeated exposure. Further, we outline key molecular changes underlying acute and chronic alcohol administration and show alcohol's effects on chromatin, gene and transcript expression, and alternative splicing vary among brain regions. Our findings indicate the extent of exposure profoundly impacts alcohol's transcriptional outcomes in the ventral hippocampus (vHPC), suggesting a robust remodeling of gene and transcript expression programs in this region during the transition from acute to chronic ethanol consumption. Overall, this study systematically illuminates key epigenetic and transcriptomic outcomes linked to ethanol and outlines how these changes differ between acute and chronic exposure, which will guide the development of future therapeutic interventions.

## Materials and Methods

### Animals and experimental design

Animal use and all experiments performed were approved by the Institutional Animal Care and Use Committee (IACUC protocol ID 22-0418). All personnel involved have been adequately trained and are qualified according to the Animal Welfare Act (AWA) and the Public Health Service (PHS) policy. C57BL/6J male mice were obtained from The Jackson Laboratory (RRID:IMSR_JAX:00064) and were group housed on a 12 h light/dark cycle with food and water provided *ad libitum*. Male C57BL/6J mice 8–10 weeks of age were used for all experiments. Mice were randomly assigned to treatment groups.

**Figure 1.**
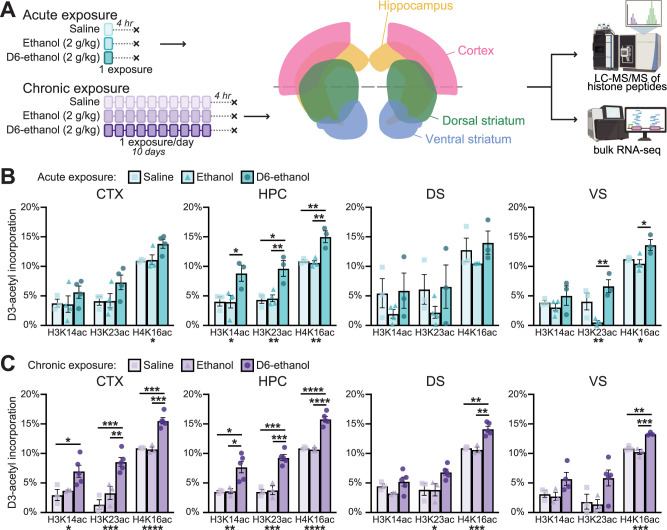
Ethanol-derived acetate contributes to histone acetylation in brain regions relevant to AUD following acute or chronic exposure. ***A***, Experimental workflow showing acute and chronic ethanol exposure models, coronal view of investigated brain regions, and downstream molecular assays. ***B***, ***C***, Proportion of heavy labeled (D3-)acetyl incorporation into histone acetylation in brain regions of interest following (***B***) acute exposure and (***C***) chronic exposure. Data are means ± SEM, with symbols showing individual mice. *n* = 3–5 mice per group. Asterisks denote significance level (*<0.05, **<0.01, ***<0.001, ****<0.0001) of *p* value from one-way ANOVA (below *x*-axis) or adjusted *p* value from post hoc Tukey's multiple-comparisons test (above columns). Abbreviations: CTX, cortex; DS, dorsal striatum; HPC, whole hippocampus; VS, ventral striatum. See Extended Data Figure 1-1 for heavy labeling data and statistical reports.

Each mouse was administered with either saline (vehicle control), 2 g/kg ethanol (20% ethanol in saline solution), or 2 g/kg heavy labeled ethanol (20% deuterated [D6-]ethanol in saline solution) via intraperitoneal (i.p.) injection. Mice acutely exposed were administered 1 dose, whereas mice chronically exposed were administered 10 doses over the course of 10 consecutive days (1 dose per day; Fig. 1*A*). Mice were killed 4 h postinjection by cervical dislocation. Whole brains were immediately collected in cryogenic vials, snap-frozen in a bath of 2-methylbutane (Sigma-Aldrich, M32631) embedded in dry ice, and stored at −80°C until dissection of brain regions. Brain tissue was dissected on a −20°C cold plate, using a razor blade to make coronal slices and 11-gauge sample corer (Fine Science Tools, 180–35) to collect specific brain regions from coronal slices. Collected frozen tissue was stored at −80°C until used for one of the two downstream molecular assays used in this study (mass spectrometry or RNA sequencing). Mass spectrometry samples were derived from *n* = 3–5 mice per group, with groups defined by paradigm (acute or chronic) and treatment (saline, ethanol, or D6-ethanol; 6 groups; *n* = 22 mice total). RNA sequencing samples were derived from *n* = 3–4 mice per group, with groups defined by paradigm (acute or chronic) and treatment (saline or ethanol; 4 groups; *n* = 16 mice total).

10.1523/ENEURO.0484-25.2026.f1-1 Figure 1-1 **Data and statistical reports for heavy acetyl incorporation into histone acetylation. (A)** Heavy LC-MS/MS data of D3-acetyl incorporation into investigated histone peptides in the context of acute or chronic exposure. **(B)** Statistical reports from analyses of heavy LC-MS/MS data. Supports ***Figure 1B,C***. Download Figure 1-1, XLS file.

### Histone preparation and mass spectrometry

Histone extraction and sample preparation for mass spectrometry were performed as previously described (Sidoli et al., 2016). A liquid chromatography-tandem mass spectrometry (LC-MS/MS) system consisting of a Vanquish Neo UHPLC coupled to an Orbitrap Q Exactive or Ascend (Thermo Scientific) was used for peptide analysis. Histone peptide samples were maintained at 7°C on a sample tray in LC. Separation of peptides was carried out on an Easy-Spray PepMap Neo nano-column (2 µm, C18, 75 µm × 150 mm) at room temperature with a mobile phase. The chromatography conditions consisted of a linear gradient from 2–32% solvent B (0.1% formic acid in 100% acetonitrile) in solvent A (0.1% formic acid in water) over 48 min and then 42–98% solvent B over 12 min at a flow rate of 300 nl/min. The mass spectrometer was programmed for data-independent acquisition (DIA). One acquisition cycle consisted of a full MS scan and 35 DIA MS/MS scans of 24 m/z isolation width starting from 295 to 1,100 m/z. Full MS scans were typically acquired in the Orbitrap mass analyzer across 290–1,200 m/z at a resolution of 70,000 or 120,000 in positive profile mode with an injection time of 50 ms and an automatic gain control (AGC) target of 1.0 × 10^−6^ or 200%. MS/MS data from higher energy collisional dissociation fragmentation was collected in the Orbitrap. These scans typically used a nominal collision energy of 30 or 25, AGC target of 1,000%, and maximum injection time of 60 ms. EpiProfile 3 (Yuan et al., 2015) was used to analyze histone MS data and calculate the ratio at which each modification was present for each peptide (relative abundance).

### RNA extraction and sequencing

Total RNA was extracted using TRIzol-chloroform and the RNeasy Mini Kit (Qiagen, 74104) with on-column RNase-free DNase (Qiagen, 79254) treatment according to manufacturer protocols, and total RNA quality was assessed using High Sensitivity RNA ScreenTape Analysis on the TapeStation system (Agilent, 5067-5579 and 5067-5580).

mRNA was isolated from up to 100 ng total RNA using the NEBNext Poly(A) mRNA Magnetic Isolation Module (New England Biolabs, E7490), and libraries were prepared using the NEBNext Ultra II RNA Library Prep Kit for Illumina (New England Biolabs, E7770; for cortex and dHPC) or the NEBNext Ultra II Directional RNA Library Prep Kit for Illumina (New England Biolabs, E7760; for vHPC, DS, and VS) in coordination with compatible NEBNext Multiplex Oligos for Illumina (New England Biolabs, E7335, E7500, and E7710 for cortex and dHPC; E6440 for vHPC, DS, and VS) and HighPrep PCR beads (MagBio, AC-60050). Library quality was assessed using DNA ScreenTape Analysis on the TapeStation system (Agilent, 5067-5602 and 5067-5582). Libraries were quantified using the NEBNext Library Quant Kit (New England Biolabs, E7630) with an Applied Biosystems QuantStudio 5 Real-Time PCR System. The NEBioCalculator qPCR Library Quantitation Tool was used to calculate the concentration of amplifiable templates in each library. Following quantitation, libraries were pooled in equal concentrations and submitted for sequencing.

Libraries for the cortex and dHPC were sequenced on the AVITI system using the AVITI 2 × 75 Sequencing Kit Cloudbreak Freestyle High Output Kit (Element Biosciences, 860-00015), and base calls and demultiplexing were performed using Element Biosciences' bases2fastq software allowing 1 mismatch in the indexing read at The DNA Sequencing Innovation Lab (DSIL) at The Center for Genome Sciences and Systems Biology (CGSSB) at Washington University School of Medicine in St. Louis. Libraries for the vHPC, DS, and VS were sequenced on an Illumina NovaSeq 6000, and base calls and demultiplexing were performed with Illumina's bcl2fastq software and a custom python demultiplexing program with a maximum of 1 mismatch in the indexing read at The Genome Technology Access Center (GTAC) at The McDonnell Genome Institute (MGI) at Washington University School of Medicine in St. Louis. Sequencing generated ∼30 million paired-end reads on average per sample (2 × 75 bp for cortex and dHPC; 2 × 150 bp for vHPC, DS, and VS).

### RNA-seq analysis and visualization

Adapters were trimmed from sequencing reads using Cutadapt v5.0 (Martin, 2011) with a minimum length of 15 bp (parameters “-a AGATCGGAAGAGCACACGTCTGAACTCCAGTCA -A AGATCGGAAGAGCGTCGTGTAGGGAAAGAGTGT -m 15”). Trimmed reads were aligned to the *Mus musculus* GRCm39 reference genome assembly using STAR v2.7.10a (Dobin et al., 2013) with parameters “–runMode alignReads –outSAMtype BAM SortedByCoordinate –outFilterType BySJout –outFilterMultimapNmax 20 –alignSJoverhangMin 8 –alignSJDBoverhangMin 1 –outFilterMismatchNmax 999 –outFilterMismatchNoverLmax 0.04 –alignIntronMin 20 –alignIntronMax 100,000 –alignMatesGapMax 100,000 –quantMode TranscriptomeSAM GeneCounts”. Gene and transcript isoform counts were generated using featureCounts v2.0.6 (Liao et al., 2014) and the annotation from the NCBI RefSeq assembly (Mus_musculus.GRCm39.113.gtf) with parameters “-t exon -g gene_id -s 2 -p -B -C -O -M” for gene counts and “-t exon -g transcript_id -s 2 -p -B -C -O -M” for transcript isoform counts.

Before differential expression analysis (DEA), low counts were filtered out following the criteria that at least two samples in a group have at least five counts each per row (groups defined by paradigm, treatment, and region). Differential gene and transcript isoform expression analyses were separately performed, with DESeq2 (Love et al., 2014) using the default Wald negative binomial test to determine differentially expressed genes (DEGs) or differentially expressed transcripts (DETs) for pairwise comparisons (ethanol vs saline, by paradigm and region). For each region, interaction effects between paradigm and treatment were evaluated with DESeq2 (Love et al., 2014) using “design = ∼Paradigm*Trt” and statistical testing for the contrast “name = ‘ParadigmChronic.TrtEtOH’”. A false discovery rate (FDR) cutoff of 0.1 and no log_2_(fold change; LFC) cutoff was used in differentiating differentially expressed from nondiffering genes or transcripts. RNA-seq differential expression and interaction analysis results are shown in Extended Data Figure 3-1 for gene level and Extended Data Figure 5-1 for transcript isoform level. Unless otherwise specified, the R package ggplot2 (Wickham et al., 2016) was used to generate plots of RNA-seq data.

Alternative splicing was assessed using rMATS v4.3.0 (Shen et al., 2014) with variable read lengths and soft-clipped reads permitted (parameters “–variable-read-length –allow-clipping”, with “–readLength 79 –libType fr-unstranded” for cortex and dHPC or “–readLength 151 –libType fr-firststrand” for vHPC, DS, and VS) and the annotation from the NCBI RefSeq assembly (Mus_musculus.GRCm39.113.gtf). Differential alternative splicing events (ASEs) at annotated splice junctions were identified between samples from ethanol- versus saline-treated groups (within-paradigm and -region), with difference in percent spliced in (ΔPSI) calculated as ethanol minus saline. Significant ASEs were distinguished by FDR < 0.1 and |ΔPSI| ≥ 0.1. rMATS results are shown in Extended Data Figure 5-4.

Gene ontology (GO) enrichment tests were performed for biological process terms with at least 10 annotated genes (“ontology = ‘BP’, nodeSize = 10”) using the weight01 algorithm and Fisher statistic (“algorithm = ‘weight01’, statistic = ‘fisher’”) with the R package topGO (Alexa and Rahnenfuhrer, 2024). Resulting *p*-values were adjusted using Benjamini–Hochberg correction, and significantly enriched terms were distinguished by FDR < 0.1. When >10 significant terms were identified, redundancy was reduced using the R package rrvgo (Sayols, 2023) as follows: Semantic similarity between terms was calculated, a similarity matrix was generated (Lin method), terms were clustered (similarity threshold specified in corresponding Extended Data), and representative nonredundant terms (one parent term per cluster) were retained. For each term, gene ratio was calculated as the number of significant genes out of the number of annotated genes detected. GO enrichment results are shown in Extended Data Figures 3-2, 4-1, 5-3, and 6-1.

Given differences in library preparation and sequencing platforms between brain regions, quantitative analyses were limited to within-region comparisons and only qualitative and categorical summaries were used to make comparisons between regions. The list of queried response genes (RGs) used for plotting Extended Data Figure 3-3 was curated based on published literature (Vazdarjanova et al., 2002; Chen et al., 2004; Schmahl et al., 2007; Tullai et al., 2007; Kojima et al., 2008; Trollmann et al., 2010; Saha et al., 2011; Saha and Dudek, 2013; Zovkic et al., 2014; Dunn et al., 2017; Kim et al., 2018; Tyssowski et al., 2018; Periandri et al., 2026) and is provided in Extended Data Figure 3-1*B*.

### Statistical analyses

Sample sizes and statistical tests are indicated in the figure legends. Bar plots with error bars represent means ± SEM, and bar plots without error bars represent total quantities. Boxplots represent median (center line), interquartile range (box edges), and range (whiskers). Shapes (datapoints) on bar and boxplots represent individual mice.

Heavy labeled LC-MS/MS data were analyzed using Skyline (MacLean et al., 2010). The relative amount of heavy labeling was calculated as the peak area of the heavy (D3-labeled) peptide divided by the sum of the peak areas of the heavy and light peptides (data in Extended Data Fig. 1-1*A*). D6-ethanol-derived acetyl groups were most reliably detected for H3K14ac, H3K23ac, and H4K16ac due to these peptides being abundantly acetylated. Thus, we chose to focus on these three hPTMs to assess D3-acetyl incorporation. Heavy labeled LC-MS/MS data were statistically analyzed in GraphPad Prism to test for normal distribution (Shapiro–Wilk test), test for significant treatment effect (one-way ANOVA for parametric data, Kruskal–Wallis test for nonparametric data), and in cases of significant treatment effect, post hoc test for pairwise differences between treatment groups (Tukey's multiple-comparisons test for parametric data, Dunn's multiple-comparisons test for nonparametric data). Statistical reports for heavy labeled LC-MS/MS data are in Extended Data Figure 1-1*B*.

Unlabeled LC-MS/MS data were captured as histone peptide relative abundance ratios for samples from ethanol- and saline-treated mice (i.e., excluding samples from D6-ethanol-treated mice). For unlabeled LC-MS/MS data, initial pairwise statistical analyses were performed in Excel using Student's *t* test (unpaired, two-tailed, equal variance) for each histone peptide (per row) to compare ethanol- versus saline-treated groups within a cohort (*p* values reported in Extended Data Fig. 2-1*B*). Unlabeled LC-MS/MS data were further analyzed in R using two-way ANOVA to detect significant paradigm × treatment interaction or main effect of either factor (*p < 0.05 indicated by row annotation on heatmaps, statistical reports in Extended Data Fig. 2-1*C*). In cases of a significant interaction, post hoc pairwise comparisons between treatments (within-paradigm) were tested using estimated marginal means [EMMs, with the R package emmeans (Lenth and Piaskowski, 2025)] with the AOV model and Benjamini–Hochberg correction for multiple comparisons per histone peptide (*p*-adj. significance level shown as asterisks on heatmaps, statistical reports in Extended Data Fig. 2-1*D*).

### Data accessibility

All mass spectrometry and RNA sequencing data are publicly available at the time of publication. Mass spectrometry data have been deposited in the MassIVE Repository (accession: MSV000100736). RNA sequencing data have been deposited in the NCBI Gene Expression Omnibus (GEO; accession: GSE31424).

## Results

### Ethanol-derived acetate is incorporated into brain histone acetylation following acute or chronic exposure

To determine whether acetate produced during ethanol metabolism directly contributes to histone acetylation in key substance use-related regions of the mouse brain, we used stable isotope-labeling mass spectrometry targeting histone peptides (Mews and Berger, 2016) in the cortex, whole hippocampus (HPC), dorsal striatum (DS), and ventral striatum (VS). Adult male C57BL/6J mice were administered a daily intraperitoneal (i.p.) injection of 2 g/kg heavy labeled ethanol (D6-ethanol), 2 g/kg ethanol, or equivalent volume saline for 1 d (acute exposure) or 10 d (chronic exposure) and were killed 4 h following the final exposure (Fig. 1*A*). Due to their generally high abundance and reliable detectability, we focused our analysis on H3K14ac, H3K23ac, and H4K16ac peptides.

Supporting our previous findings (Mews et al., 2019), acute treatment significantly affected the fraction of deuterated D3-acetyl of all three investigated peptides in the HPC (one-way ANOVA: [H3K14ac] *F*_(2,7)_ = 6.096, *p* = 0.0293; [H3K23ac] *F*_(2,7)_ = 4.11, *p* = 0.0063; [H4K16ac] *F*_(2,7)_ = 17.75, *p* = 0.0018; data and statistical reports in Extended Data Fig. 1-1), with significantly higher D3-acetyl incorporation in the acute D6-ethanol group compared with other acute treatment groups (Fig. 1*B*; post hoc Tukey's multiple comparisons reported in Extended Data Fig. 1-1*B*). In contrast, acute treatment affected D3-acetyl incorporation only for H4K16ac in the cortex (one-way ANOVA: [H4K16ac] *F*_(2,8)_ = 5.145, *p* = 0.0366; [H3K14ac] *F*_(2,8)_ = 1.000, *p* = 0.4095; [H2K23ac] *F*_(2,8)_ = 2.559, *p* = 0.1383), with no significant pairwise differences between treatment groups (Fig. 1*B*; post hoc reported in Extended Data Fig. 1-1*B*). Further emphasizing brain region specificity, no peptide was affected in the DS (Fig. 1*B*; one-way ANOVA: [H3K14ac] *F*_(2,7)_ = 1.197, *p* = 0.3572; [H3K23ac] *F*_(2,7)_ = 1.101, *p* = 0.3840; [H4K16ac] *F*_(2,6)_ = 1.222, *p* = 0.3588), while in the VS, acute exposure significantly affected D3-acetyl incorporation into H3K23ac and H4K16ac (one-way ANOVA: [H3K23ac] *F*_(2,7)_ = 11.35, *p* = 0.0064; [H4K16ac] *F*_(2,7)_ = 6.287, *p* = 0.0273; [H3K14ac] *F*_(2,7)_ = 1.094, *p* = 0.3860), with significantly higher incorporation in the acute D6-ethanol group (Fig. 1*B*; post hoc reported in Extended Data Fig. 1-1*B*). Together, these findings indicate a single exposure to ethanol is sufficient for ethanol-derived acetate incorporation into histone acetylation in some (HPC and VS) but not all (cortex or DS) investigated brain regions.

We next assessed the extent of ethanol-derived acetate incorporation into histone acetylation in these brain regions following chronic exposure, which has previously not been investigated. Chronic treatment significantly affected the fraction of D3-acetyl incorporation into each investigated peptide in both the HPC and cortex (one-way ANOVA: [H3K14ac] HPC *F*_(2,8)_ = 9.213, *p* = 0.0085 and cortex *F*_(2,8)_ = 5.769, *p* = 0.0281; [H3K23ac] HPC *F*_(2,8)_ = 35.72, *p* < 0.0001 and cortex *F*_(2,8)_ = 19.74, *p* = 0.0008; [H4K16ac] HPC *F*_(2,8)_ = 56.59, *p* < 0.0001 and cortex *F*_(2,8)_ = 37.12, *p* < 0.0001), with significantly higher incorporation in the chronic D6-ethanol groups (Fig. 1*C*; post hoc Tukey's multiple comparisons reported in Extended Data Fig. 1-1*B*). In the DS, chronic treatment significantly affected D3-acetyl incorporation into H3K23ac and H4K16ac (one-way ANOVA: [H3K23ac] *F*_(2,8)_ = 5.555, *p* = 0.0307; [H4K16ac] *F*_(2,8)_ = 22.21, *p* = 0.0005; [H3K14ac] *F*_(2,8)_ = 2.317, *p* = 0.1608), with the chronic D6-ethanol group showing significantly higher D3-acetyl incorporation into H4K16ac but not H3K23ac (Fig. 1*C*; post hoc reported in Extended Data Fig. 1-1*B*). Finally, we found chronic treatment significantly affected D3-acetyl incorporation only for H4K16ac in the VS (one-way ANOVA: [H4K16ac] *F*_(2,6)_ = 29.37, *p* = 0.0008; [H3K14ac] *F*_(2,7)_ = 3.288, *p* = 0.0985; [H3K23ac] *F*_(2,8)_ = 3.971, *p* = 0.0634), and the chronic D6-ethanol group had significantly higher D3-acetyl incorporation into H4K16ac in comparison to the other chronic treatment groups (Fig. 1*C*; post hoc Tukey's multiple comparisons reported in Extended Data Fig. 1-1*B*). Overall, these findings indicate ethanol-derived acetate is incorporated into histone acetylation following chronic exposure, with a broader and more robust impact on brain regions relevant to AUD and acetylated peptides compared with acute exposure. Interestingly, our results suggest different brain regions may have differing levels of sensitivity to the epigenetic impact of ethanol-derived acetate.

### Ethanol exposure affects histone posttranslational modifications in a brain region-specific manner

To understand the general impact of acute or chronic ethanol exposure on histone posttranslational modifications (hPTMs) in these key substance use-related brain regions, we performed LC-MS/MS targeting histone peptides in male mice that were administered a daily dose of 2 g/kg ethanol or equivalent volume saline via intraperitoneal injection for 1 d (acute) or 10 d (chronic) and killed 4 h following the final injection (Fig. 1*A*).

**Figure 2.**
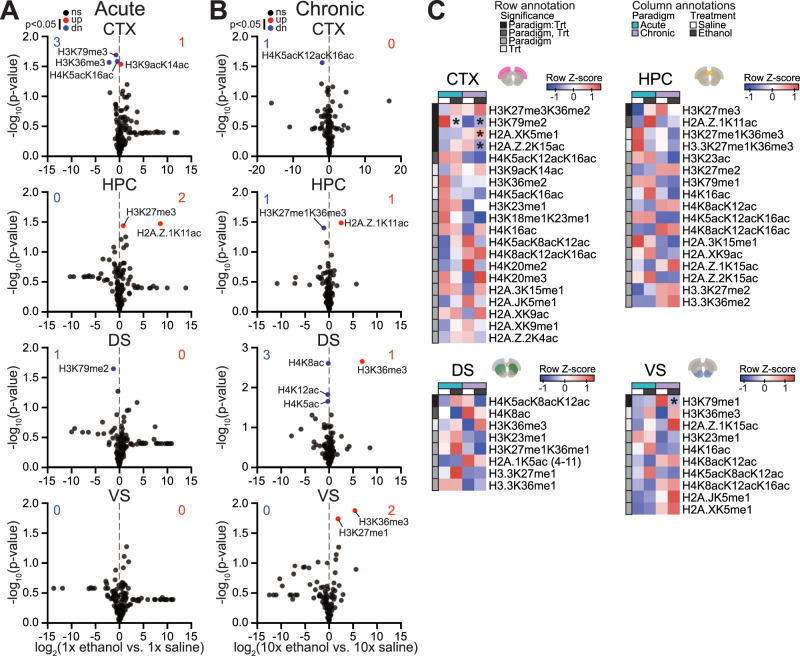
Chromatin remodeling in AUD-related brain regions following acute or chronic ethanol exposure. ***A***, ***B***, Volcano plots of hPTMs comparing ethanol- versus saline-treated mice following (***A***) acute exposure and (***B***) chronic exposure for each region. ***C***, Heatmaps showing average row *Z*-score of relative abundance for hPTMs showing a significant paradigm-dependent change in response to ethanol treatment (paradigm × treatment interaction [Paradigm:Trt]) or main effect of paradigm (chronic vs acute), treatment (ethanol vs saline [Trt]), or both factors (Paradigm, Trt; *p* < 0.05 by two-way ANOVA, indicated by row annotation). *p-adj. < 0.05 (estimated marginal means with the AOV model and Benjamini–Hochberg multiple comparisons correction). *n* = 3–4 mice per group. Abbreviations: dn, down; ns, not significant. See Extended Data Figure 2-1 for histone ratio data and statistical reports.

Acute ethanol exposure significantly affected the average relative abundance of hPTMs in each investigated region except the VS (Fig. 2*A*; data and statistical reports in Extended Data Fig. 2-1). The most hPTM changes in response to acute ethanol exposure were observed in the cortex, where H3K9acK14ac was increased while H4K5acK16ac, H3K36me3, and H3K79me3 were reduced (Fig. 2*A*). Dorsal striatal H3K79me2 was significantly reduced, whereas hippocampal H3K27me3 and H2A.Z.1K11ac were significantly increased following acute ethanol exposure (Fig. 2*A*).

10.1523/ENEURO.0484-25.2026.f2-1 Figure 2-1 **Data and statistical reports for histone marks. (A)** Relative abundance of histone peptides (histone ratios). **(B)** *P*-values from two-tailed t test of equal variance comparing treatment (ethanol vs. saline) within each paradigm for histone peptides (*p*<0.05 indicated by red or blue dot color in ***Figure 2A,B***). **(C)** Two-way ANOVA results for hPTMs with significant paradigm-dependent change in response to ethanol treatment (paradigm × treatment interaction [Paradigm:Trt]) or main effect of paradigm (chronic vs. acute), treatment (ethanol vs. saline [Trt]), or both factors (*p*<0.05 indicated by row annotation in ***Figure 2C***). **(D)** Post hoc pairwise comparisons of treatment (ethanol vs. saline) within each paradigm for hPTMs with significant paradigm × treatment interaction (*p*<0.05 by two-way ANOVA). Pairwise tests were performed using estimated marginal means with the aov model, and *p*-values were adjusted for multiple comparisons using the Benjamini-Hochberg method (significance level indicated by asterisks in ***Figure 2C***). Supports ***Figure 2***. Download Figure 2-1, XLS file.

Chronic ethanol exposure also significantly affected the average relative abundance of hPTMs in each investigated region (Fig. 2*B*). In contrast to acute exposure, the most hPTM changes were observed in the DS, where H4K5ac, H4K8ac, and H4K12ac were reduced while H3K36me3 was increased (Fig. 2*B*). Ventral striatal H3K36me3 was also significantly increased in response to chronic ethanol exposure, as was H3K27me1 (Fig. 2*B*). In contrast to acute exposure, chronic ethanol exposure affected only one hPTM in the cortex: H4K5acK12acK16ac was reduced (Fig. 2*B*). In the HPC, H3K27me1K36me3 was reduced and H2A.Z.1K11ac was increased in response to chronic ethanol exposure (Fig. 2*B*).

The average relative abundance of only one hPTM (H3K36me3) was significantly affected in more than one region in response to ethanol exposure, indicating ethanol-induced hPTM changes occur primarily in a brain region-specific manner. Further, only two hPTMs (H3K36me3 and H2A.Z.1K11ac) were affected in both exposure paradigms, raising the possibility that ethanol-induced hPTM changes are dependent upon the extent of ethanol exposure. To test this, we performed two-way ANOVA analyses within each region to identify hPTMs with a significant interaction between paradigm (acute or chronic) and treatment (ethanol or saline) or a main effect of either factor. In the cortex, four hPTMs were differentially influenced by ethanol depending on the extent of exposure: H3K27me3K36me2, H3K79me2, H2A.XK5me1, and H2A.Z.2K15ac (Fig. 2*C*; two-way ANOVA reported in Extended Data Fig. 2-1*C*). Acute ethanol exposure significantly decreased H3K79me2 abundance but did not significantly affect the abundance of the other hPTMs, whereas chronic ethanol exposure significantly increased H2A.XK5me1 and H3K79me2 and reduced H2A.Z.2K15ac abundance in the cortex (Fig. 2*C*; post hoc reported in Extended Data Fig. 2-1*D*). For each of the other investigated regions, only one hPTM was differentially influenced by ethanol in a paradigm-dependent manner: H3K27me3 in the HPC (two-way ANOVA, paradigm × treatment interaction: *F*_(1,9)_ = 8.129, *p* = 0.0191), H4K5acK8acK12ac in the DS (*F*_(1,9)_ = 8.6322, *p* = 0.0165), and H3K79me1 in the VS (*F*_(1,9)_ = 5.410, *p* = 0.0450; Fig. 2*C*). Of these, H3K79me1 was reduced in the VS in response to chronic ethanol exposure (Fig. 2*C*; post hoc reported in Extended Data Fig. 2-1*D*). No hPTMs showed a significant interaction between paradigm and treatment for more than one region, further supporting regional specificity.

We also observed a paradigm-independent effect of ethanol on the epigenome of each investigated brain region, as evidenced by hPTMs showing a significant main effect of treatment. Specifically, there were four hPTMs with a significant treatment effect in the cortex (H3K9acK14ac increased; H3K36me2, H4K5acK16ac, and H4K5acK12acK16ac decreased), three in HPC (H2A.Z.1K11ac increased; H3K27me1K36me3 and H3.3K27me1K36me3 decreased), two in DS (H3K36me3 increased; H4K8ac decreased), and two in VS (H3K36me3 and H2A.Z.1K15ac increased; Fig. 2*C*; two-way ANOVA reported in Extended Data Fig. 2-1*C*). H3K36me3 was significantly increased in both the DS and VS in response to ethanol regardless of paradigm and was the only hPTM showing a significant treatment effect in more than one region (Fig. 2*C*), suggesting regional specificity of paradigm-independent epigenetic effects of ethanol.

More hPTMs exhibited a significant main effect of paradigm, suggesting a potential treatment-independent impact of repeated intraperitoneal injections on the brain epigenome. Specifically, there were 13 hPTMs with a significant paradigm effect in the cortex, 14 in HPC, 6 in DS, and 7 in VS (Fig. 2*C*; two-way ANOVA reported in Extended Data Fig. 2-1*C*). Of note, nearly a third of hPTMs significantly affected by paradigm were observed in more than one region, suggesting paradigm effects are less regionally specific than ethanol-induced hPTM changes.

Taken together, these findings indicate ethanol's effects on hPTMs depend largely on brain region and in part on extent of exposure. Importantly, acetylation and methylation changes occurred at similar frequencies, indicating the epigenetic impact of ethanol extends beyond direct incorporation of acetyl groups.

### Acute and chronic ethanol exposure disrupts gene expression in a brain region-specific manner

Next, we examined the gene expression outcomes associated with the observed epigenetic changes. We used RNA-seq to investigate the transcriptomic effects of acute or chronic ethanol exposure across five substance use-related brain regions: The cortex, dorsal hippocampus (dHPC), ventral hippocampus (vHPC), DS, and VS.

**Figure 3.**
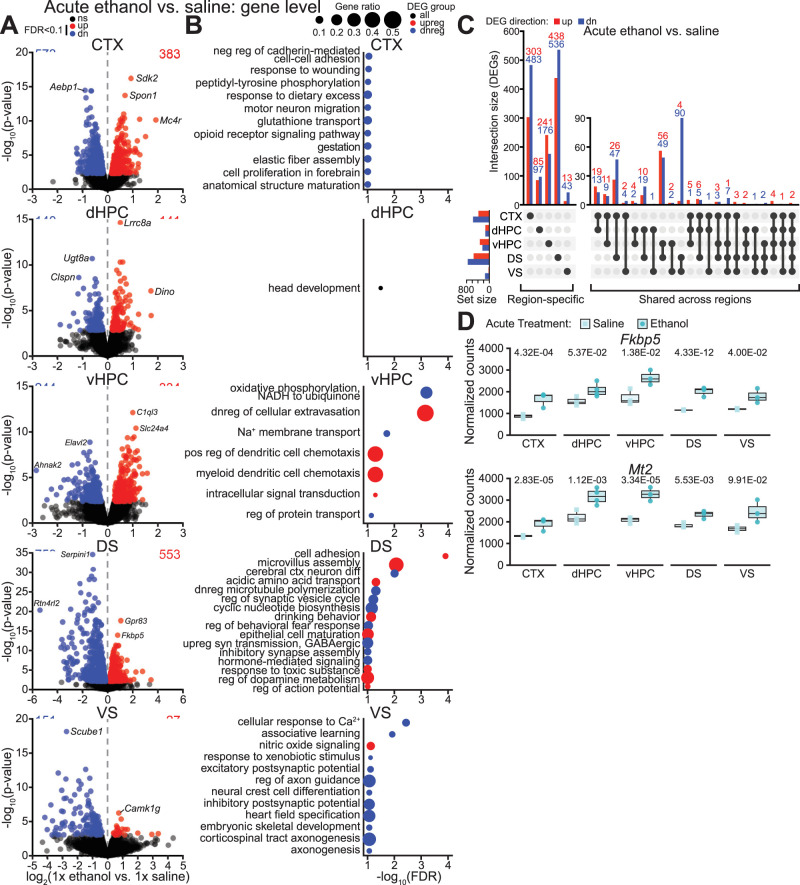
Acute ethanol exposure dysregulates gene expression in a brain region-specific manner. ***A***, Volcano plots of differential gene expression in each region following acute ethanol exposure. ***B***, Dot plots of top significantly enriched biological processes for DEGs in each region following acute ethanol exposure. ***C***, Upset plot showing overlap of DEGs across regions following acute ethanol exposure. ***D***, Normalized gene counts for *Fkbp5* and *Mt2* in each region following acute exposure with FDR from DESeq2 shown. *n* = 3–4 mice per group. Abbreviations: dnreg, downregulation; neg reg, negative regulation; pos reg, positive regulation; reg, regulation; syn, synaptic; upreg, upregulated. See Extended Data Figure 3-1 for gene level differential expression results, Extended Data Figure 3-2 for gene ontology (GO) enrichment results, and Extended Data Figure 3-3 for differential expression of response genes (RGs).

Acute ethanol exposure significantly dysregulated gene expression in each investigated region: There were 956 differentially expressed genes (DEGs) in the cortex, 281 in dHPC, 578 in vHPC, 1,312 in DS, and 178 in VS (Fig. 3*A*; differential expression analysis results in Extended Data Fig. 3-1*A*). To identify possible functional implications of these gene expression changes, we performed gene ontology (GO) enrichment analysis of biological processes for each region. Acute ethanol induced cortical downregulation of genes related to various processes including glutathione transport, opioid receptor signaling, and peptidyl-tyrosine phosphorylation (Fig. 3*B*, Extended Data Fig. 3-2*A*). Dorsal hippocampal DEGs were enriched for head development, while upregulated genes in the vHPC were enriched for processes involving dendritic cell chemotaxis and downregulated genes in the vHPC were associated with regulation of protein transport (Fig. 3*B*, Extended Data Fig. 3-2*B,C*). In the DS, genes upregulated by acute ethanol exposure were enriched for functions related to cell adhesion and regulation of dopamine metabolism, while downregulated genes were enriched for synaptic processes (e.g., inhibitory synapse assembly, regulation of synaptic vesicle cycle, upregulation of GABAergic synaptic transmission; Fig. 3*B*, Extended Data Fig. 3-2*D*). Genes downregulated in the VS were enriched for processes such as associative learning, axonogenesis, and postsynaptic potential (excitatory and inhibitory; Fig. 3*B*, Extended Data Fig. 3-2*E*).

10.1523/ENEURO.0484-25.2026.f3-1 Figure 3-1 **Gene level results from RNA-seq. (A)** Gene level differential expression results for pairwise comparisons (ethanol vs. saline) for each paradigm and paradigm × treatment interaction analysis. **(B)** List of queried response genes (RGs). Supports ***Figures 3 through 6***, ***Extended Data Figure 3-3***, and ***Extended Data Figure 6-2A***. Download Figure 3-1, XLS file.

10.1523/ENEURO.0484-25.2026.f3-2 Figure 3-2 **Gene ontology enrichment for DEGs in response to acute ethanol treatment. (A-E)** Results from GO enrichment analysis for acute ethanol vs. acute saline DEGs in the **(A)** cortex, **(B)** dHPC, **(C)** vHPC, **(D)** DS, and **(E)** VS. For each comparison, GO enrichment results are reported in a direction-specific manner (i.e., for up- or downregulated DEGs); when no significant direction-specific enrichments were detected, results for all DEGs (irrespective of direction) are also reported. For comparisons with >10 significantly enriched biological processes, additional columns are provided for the reduced set of non-redundant terms and the similarity threshold is specified. Supports ***Figure 3B***. Download Figure 3-2, XLS file.

10.1523/ENEURO.0484-25.2026.f3-3 Figure 3-3 **Differential expression of response genes in response to ethanol treatment.** Dot plot of response gene (RG) DEGs for each region following acute or chronic ethanol exposure. n=3-4 mice per group. See ***Extended Data Figure 3-1*** for gene level differential expression results and queried RGs. Download Figure 3-3, TIF file.

Across all investigated brain regions, acute ethanol induced significant upregulation of 1,241 genes and significant downregulation of 1,591 genes. Most of these were differentially expressed in a brain region-specific manner: 1,080 (87%) upregulated DEGs and 1,335 (84%) downregulated DEGs were significantly affected in only one region following acute ethanol exposure (Fig. 3*C*). Among genes differentially expressed in more than one brain region (161 upregulated and 256 downregulated), most were affected in two regions (83% of upregulated and 92% of downregulated shared genes). Few genes were differentially expressed in three regions (12% of upregulated and 8% of downregulated), and even fewer in four regions (3% of upregulated and no downregulated). Only two DEGs were shared across all investigated brain regions: *Fkbp5* (FK506 binding protein 5) and *Mt2* (metallothionein 2) were significantly upregulated in each region following acute ethanol exposure (Fig. 3*C*,*D*).

**Figure 4.**
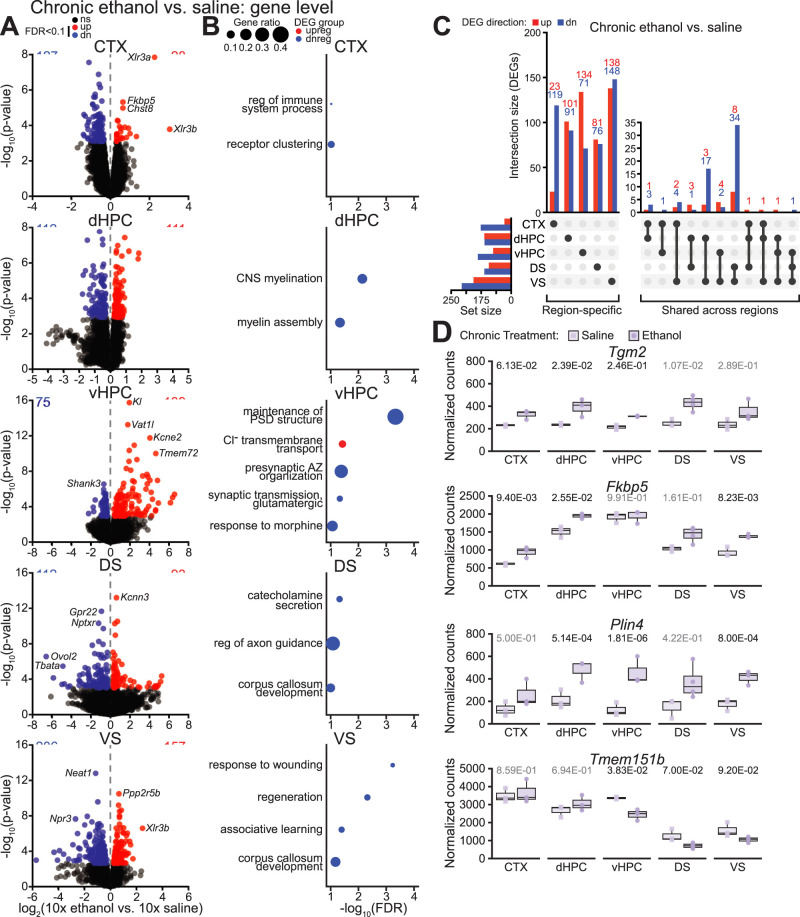
Chronic ethanol exposure dysregulates gene expression in a brain region-specific manner. ***A***, Volcano plots of differential gene expression in each region following chronic ethanol exposure. ***B***, Dot plots of top significantly enriched biological processes for DEGs in each region following chronic ethanol exposure. ***C***, Upset plot showing overlap of DEGs across regions following chronic ethanol exposure. ***D***, Normalized gene counts for *Tgm2*, *Fkbp5*, *Plin4*, and *Tmem151b* in each region following chronic exposure with FDR from DESeq2 shown. *n* = 3–4 mice per group. Abbreviations: AZ, active zone; PSD, postsynaptic density. See Extended Data Figure 3-1 for gene level differential expression results, Extended Data Figure 3-3 for differential expression of response genes (RGs), and Extended Data Figure 4-1 for GO enrichment results.

Chronic ethanol exposure induced gene expression changes in each investigated region: There were 155 DEGs in the cortex, 223 in dHPC, 214 in vHPC, 205 in DS, and 363 in VS (Fig. 4*A*; differential expression analysis results in Extended Data Fig. 3-1*A*). In the cortex, genes downregulated by chronic ethanol exposure were related to receptor clustering and regulation of immune system processes (Fig. 4*B*, Extended Data Fig. 4-1*A*). Downregulated dorsal hippocampal genes were enriched for myelination-related processes, while downregulated ventral hippocampal genes were enriched for synaptic processes and response to morphine (Fig. 4*B*, Extended Data Fig. 4-1*B,C*). Genes downregulated in the DS were enriched for catecholamine secretion and regulation of axon guidance, while genes downregulated in the VS were enriched for associative learning (Fig. 4*B*, Extended Data Fig. 4-1*D,E*).

10.1523/ENEURO.0484-25.2026.f4-1 Figure 4-1 **Gene ontology enrichment for DEGs in response to chronic ethanol treatment. (A-E)** Results from GO enrichment analysis for chronic ethanol vs. acute saline DEGs in the **(A)** cortex, **(B)** dHPC, **(C)** vHPC, **(D)** DS, and **(E)** VS. For each comparison, GO enrichment results are reported in a direction-specific manner (i.e., for up- or downregulated DEGs). For comparisons with >10 significantly enriched biological processes, additional columns are provided for the reduced set of non-redundant terms and the similarity threshold is specified. Supports ***Figure 4B***. Download Figure 4-1, XLS file.

Overall, there were fewer DEGs following chronic ethanol exposure compared with acute ethanol exposure. Across all investigated regions, chronic ethanol induced significant upregulation of 501 genes and downregulation of 568 genes. As in the case of acute exposure, most of these DEGs were specific to one brain region: 477 (95%) upregulated DEGs and 505 (90%) downregulated DEGs were significantly affected in only one region (Fig. 4*C*). Among genes differentially expressed in more than one region (24 upregulated and 63 downregulated) following chronic ethanol exposure, most were affected in two regions (88% of upregulated and 98% of downregulated shared genes) with the remaining four genes differentially expressed in three regions. Specifically, *Tgm2* (transglutaminase 1, C polypeptide) was upregulated in the cortex, dHPC, and DS; *Fkbp5* was upregulated in the cortex, dHPC, and VS; *Plin4* (perilipin 4) was upregulated in the dHPC, vHPC, and VS; and *Tmem151b* (transmembrane protein 151B) was downregulated in the vHPC, DS, and VS (Fig. 4*D*). No DEGs were shared among four or all five regions following chronic ethanol exposure.

Taken together, these findings indicate stark regional specificity of gene expression changes in the brain in response to ethanol exposure for both acute and chronic exposure paradigms. Further, acute ethanol exposure resulted in more robust gene expression dysregulation compared with chronic ethanol exposure. Notably, transient acute exposure-induced expression changes involved response genes (RGs), which were far less dysregulated after chronic ethanol exposure (Extended Data Fig. 3-3). RGs orchestrate activity-dependent plasticity and are a key component of drug-induced transcriptional changes (recently reviewed in Brida and Day, 2025).

### Ethanol-induced dysregulation of transcript expression and alternative splicing are brain region specific

Recent evidence highlights a critical connection between chronic alcohol consumption and aberrant alternative splicing (AS) in the brain (Van Booven et al., 2021; Carvalho and Lasek, 2024; Bhatnagar and Heller, 2025). However, few studies have examined AS in the context of acute ethanol exposure. To this end, we performed transcript isoform-level differential expression analyses and replicate Multivariate Analysis of Transcript Splicing (rMATS; Shen et al., 2014).

**Figure 5.**
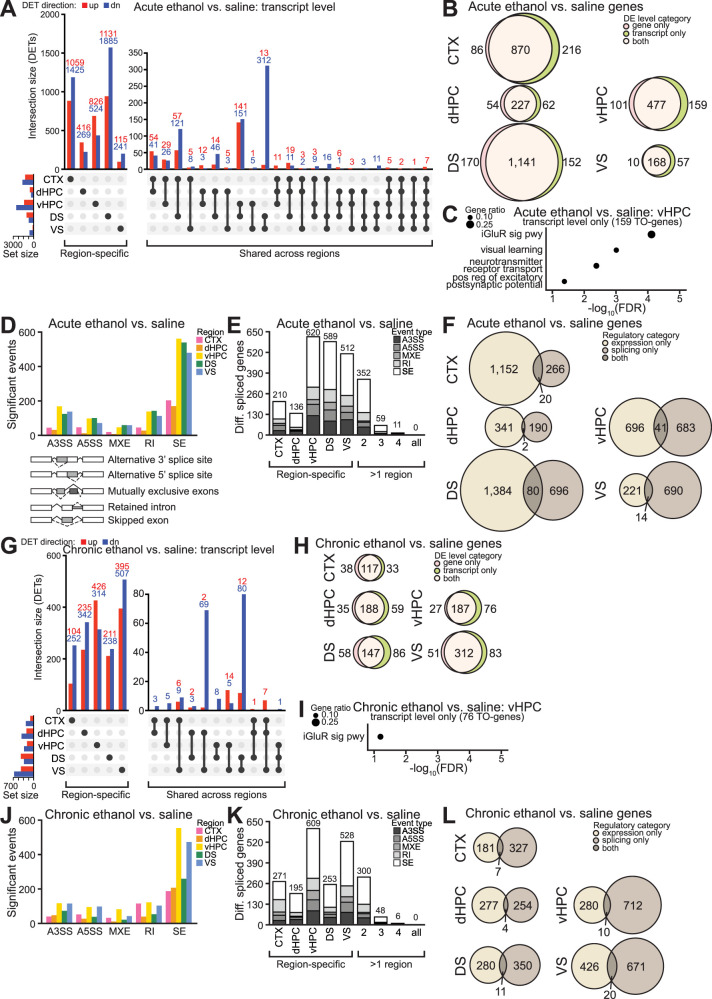
Ethanol perturbs transcript expression and alternative splicing in a brain region-specific manner. ***A***, Upset plot showing overlap of DETs across regions following acute ethanol exposure (see Extended Data Fig. 5-2*A* for per-region volcano plots). ***B***, Venn diagrams showing overlap of genes with DE at gene and transcript levels following acute ethanol exposure. ***C***, Dot plot of significantly enriched biological processes for genes in the vHPC showing transcript level-only DE (TO-genes) following acute ethanol exposure. ***D***, Quantity of alternative splicing events (ASEs) significantly differing in each region for acute ethanol versus saline, with diagram below depicting ASE types detected by rMATS. ***E***, Quantity and region specificity of differentially spliced genes (DSGs) for each ASE type following acute ethanol exposure. ***F***, Venn diagrams showing overlap of DSGs and genes showing DE (either or both levels) following acute ethanol exposure. ***G***, Upset plot showing overlap of DETs across regions following chronic ethanol exposure (see Extended Data Fig. 5-2B for per-region volcano plots). ***H***, Venn diagrams showing overlap of genes showing DE at gene and transcript levels following chronic ethanol exposure. ***I***, Dot plot of significantly enriched biological processes for TO-genes in the vHPC following chronic ethanol exposure. ***J***, Quantity of ASEs significantly differing in each region for chronic ethanol versus saline. ***K***, Quantity and region specificity of DSGs for each ASE type following chronic ethanol exposure. ***L***, Venn diagrams showing overlap of DSGs and genes showing DE (either or both levels) following chronic ethanol exposure. *n* = 3–4 mice per group. Abbreviations: A3SS, alternative 3′ splice site; A5SS, alternative 5′ splice site; DE, differential expression; diff., differentially; MXE, mutually exclusive exons; RI, retained intron; SE, skipped exon. See Extended Data Figure 5-1 for transcript level differential expression results, Extended Data Figure 5-2C for a significant A3SS event of *Stat3* exon 23 in the vHPC in response to chronic ethanol treatment, Extended Data Figure 5-3 for GO enrichment results, and Extended Data Figure 5-4 for alternative splicing results.

Acute ethanol exposure dysregulated transcript isoform expression throughout the brain: There were 2,914 differentially expressed transcripts (DETs) in the cortex, 936 in dHPC, 3,965 in vHPC, 1,775 in DS, and 756 in VS (Fig. 5*A*, Extended Data Fig. 5-2*A*; differential expression analysis results in Extended Data Fig. 5-1). Across these regions, acute ethanol exposure resulted in significant upregulation of 3,938 transcript isoforms and significant downregulation of 5,113 isoforms (Fig. 5*A*). Similar to gene level findings, the majority of DETs were specific to one region (90% of upregulated and 85% of downregulated; Fig. 5*A*). Among DETs observed in more than one region following acute ethanol exposure (391 upregulated and 769 downregulated), most were differentially expressed in two regions (85% of upregulated and 93% of downregulated). The seven upregulated DETs shared across all five regions were all encoded by *Fkbp5*, which was one of only two DEGs upregulated in each region (Fig. 3*D*). Further underlining the robust impact of acute ethanol exposure on this gene, other *Fkbp5* isoforms were differentially expressed in multiple but not all regions in response to acute ethanol exposure. Specifically, *Fkbp5-205* was upregulated in cortex, dHPC, and DS; *Fkbp5-209* was upregulated in cortex and dHPC; *Fkbp5-210* was upregulated in each region except dHPC; and *Fkbp5-212* was upregulated in each except VS. These regional differences in specific *Fkbp5* isoforms affected further highlight the regional specificity of transcript-level changes.

**Figure 6.**
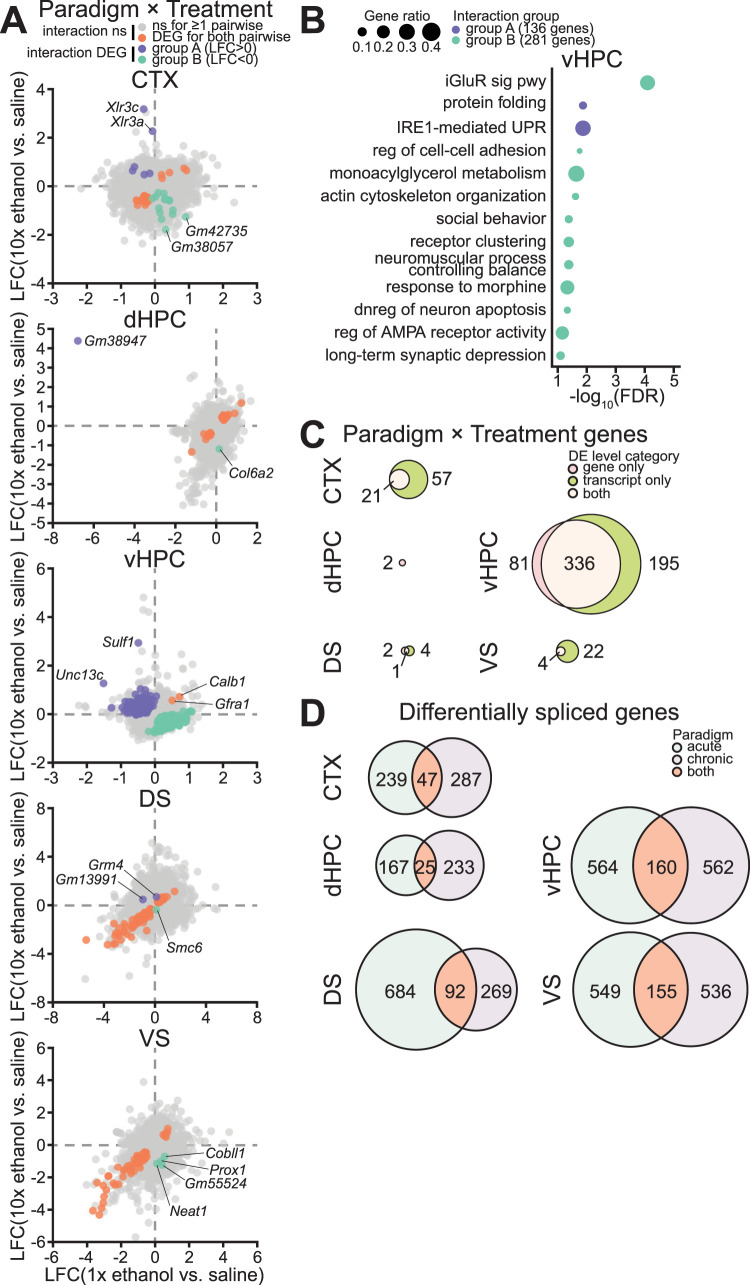
Paradigm-dependent transcriptomic effects of ethanol vary across brain regions. ***A***, Scatterplots of differential gene expression for ethanol versus saline within each paradigm, highlighting genes with significant paradigm × treatment interaction or with similar ethanol-induced effects in both paradigms (see Extended Data Fig. 6-2*A* for upset plot comparing regions). ***B***, Dot plot of significantly enriched biological processes for significant interaction genes in the vHPC. ***C***, Venn diagrams showing overlap of genes with significant interaction at gene and transcript levels (see Extended Data Fig. 6-2*B* for transcript level scatterplots and Extended Data Fig. 6-2*C* for upset plot comparing regions). ***D***, Venn diagrams showing overlap of genes with significant ethanol-induced differential AS following acute and chronic exposure. *n* = 3–4 mice per group. Abbreviations: LFC, log_2_(fold change). See Extended Data Figure 3-1 for gene level differential expression results, Extended Data Figure 6-1 for GO enrichment results, Extended Data Figure 5-1 for transcript level differential expression results, and Extended Data Figure 5-4 for alternative splicing results.

10.1523/ENEURO.0484-25.2026.f5-1 Figure 5-1 **Transcript level results from RNA-seq.** Transcript level differential expression results for pairwise comparisons (ethanol vs. saline) for each paradigm and paradigm × treatment interaction analysis. Supports ***Figures 5 and 6***, ***Extended Data Figure 5-2***, and ***Extended Data Figure 6-2B,C***. Download Figure 5-1, ZIP file.

10.1523/ENEURO.0484-25.2026.f5-2 Figure 5-2 **Transcript isoform expression and alternative splicing are dysregulated in the brain following ethanol treatment. (A-B)** Volcano plots of differential transcript expression in each brain region in response to **(A)** acute ethanol exposure and **(B)** chronic ethanol exposure. **(C)** Sashimi plot generated using rmats2sashimiplot showing a significant alternative 3' splice site (A3SS) event of *Stat3* exon 23 in the vHPC in response to chronic ethanol exposure. Genomic coordinates span *Stat3* exon 23 and the alternative 3' splice junctions corresponding to the full-length (α) and truncated (β) forms. Normalized read coverage (RPKM) is shown for each treatment group. Curved arcs represent splice junctions, with overlaid numbers denoting the average number of reads supporting the junction per sample within the group. n=3-4 mice per group. Abbreviations: ΔPSI, change in percent spliced in; RPKM, reads per kilobase million. Supports ***Figure 5***. See ***Extended Data Figure 5-1*** for transcript level differential expression results and ***Extended Data Figure 5-4*** for alternative splicing results. Download Figure 5-2, TIF file.

10.1523/ENEURO.0484-25.2026.f5-3 Figure 5-3 **Gene ontology enrichment for TO-genes. (A-C)** Results from GO enrichment analysis for acute ethanol vs. acute saline genes encoding DETs but not identified as DEGs (i.e., transcript level only genes, or TO-genes) in the **(A)** cortex, **(B)** vHPC, and **(C)** DS. **(D-F)** GO enrichment analysis results for chronic ethanol vs. chronic saline TO-genes in the **(D)** vHPC, **(E)** DS, and **(F)** VS. GO enrichment analyses of TO-genes were done in a non-direction-specific manner and were only done for comparisons with ≥75 TO-genes. Supports ***Figure 5***. Download Figure 5-3, XLS file.

10.1523/ENEURO.0484-25.2026.f5-4 Figure 5-4 **Results from alternative splicing analysis using rMATS. (A)** Alternative 3' splice site (A3SS) event results. **(B)** Alternative 5' splice site (A5SS) event results. **(C)** Mutually exclusive exons (MXE) event results. **(D)** Retained intron (RI) event results. **(E)** Skipped exon (SE) event results. Supports ***Figures 5 and 6*** and ***Extended Data Figure 5-2***. Download Figure 5-4, XLS file.

Differences between DEGs and genes encoding DETs may arise when individual transcript isoforms change in abundance but not in a manner that affects total gene expression, and functionally relevant isoform-specific changes can occur even when total gene expression does not differ. In each region, the majority of acute ethanol-induced DETs were encoded by a gene that was identified as a DEG for the respective comparison (i.e., there was differential expression at both the gene and transcript isoform levels; Fig. 5*B*). However, up to a quarter of affected genes were affected only at the transcript level (transcript level only genes, or TO-genes) in each brain region. We next performed GO enrichment analysis to query biological processes associated with TO-genes. Interestingly, the only brain region with significantly enriched biological processes linked to TO-genes was the vHPC (Extended Data Fig. 5-3*A–C*), with enriched processes including ionotropic glutamate receptor (iGluR) signaling pathway, visual learning, neurotransmitter receptor transport, and positive regulation of excitatory postsynaptic potential (Fig. 5*C*, Extended Data Fig. 5-3*B*).

AS analysis using rMATS revealed differential splicing following acute ethanol exposure was most prominent in the vHPC, subsequently followed by the DS, VS, cortex, and then dHPC (Fig. 5*D*; splicing analysis results in Extended Data Fig. 5-4). In each region, exon skipping was the most frequently observed differential AS event (ASE) type in response to acute ethanol exposure. Regardless of ASE type, the majority of differentially spliced genes (DSGs) in response to acute ethanol exposure were specific to one brain region (Fig. 5*E*), underscoring the regional specificity of transcriptomic changes elicited by acute ethanol treatment. Additionally, there was minimal overlap between DSGs and genes with expression changes: For each region, <4% of affected genes showed changes in both expression and splicing (Fig. 5*F*), suggesting acute ethanol exposure disrupts independent regulatory pathways of expression and AS.

As expected, chronic ethanol exposure also perturbed transcript expression in the brain: There were 387 DETs in the cortex, 664 in dHPC, 556 in vHPC, 773 in DS, and 1,107 in VS (Fig. 5*G*, Extended Data Fig. 5-1*B*; differential expression results in Extended Data Fig. 5-1). Similar to gene level findings, the overall impact of chronic ethanol exposure was also lesser compared with acute exposure at the transcript level. Across all regions, chronic ethanol exposure induced significant upregulation of 1,415 transcript isoforms and significant downregulation of 1,826 transcript isoforms (Fig. 5*G*). The majority of these DETs were region specific (97% of upregulated and 90% of downregulated, Fig. 5*G*) and predominantly encoded by genes identified as DEGs in response to chronic ethanol exposure (Fig. 5*H*). Again, the vHPC was the only brain region with significantly enriched biological processes associated with TO-genes (Extended Data Fig. 5-3*E–G*), specifically iGluR signaling (Fig. 5*I*, Extended Data Fig. 5-3*D*).

In response to chronic ethanol exposure, differential AS was most prominent in the vHPC, subsequently followed by the VS, DS, cortex, and then dHPC (Fig. 5*J*; splicing analysis results in Extended Data Fig. 5-4). The most frequently observed ASE type following chronic ethanol exposure was exon skipping (Fig. 5*J*), and there was substantial regional specificity of DSGs regardless of event type (Fig. 5*K*), similar to differential AS driven by acute ethanol exposure. DSGs and genes with expression changes minimally overlapped, with <2% of affected genes showing changes in both expression and splicing (Fig. 5*L*), indicating chronic ethanol exposure disrupts independent transcriptional regulatory pathways. For example, chronic ethanol treatment significantly decreased the inclusion of an alternative 3′ splice site (A3SS) event at exon 23 of the transcription factor *Stat3* (signal transducer and activator of transcription 3) in the vHPC (Extended Data Fig. 5-2*C*) but did not significantly affect the expression of this gene or any of its isoforms.

Dysregulation of transcript expression and AS were more prominent in response to acute ethanol exposure compared with chronic exposure, aligning with our gene level findings. Transcript level and AS analyses further underlined the brain region-specific nature of ethanol-induced molecular changes. Additionally, these results identified the vHPC as particularly susceptible to transcript-level disruptions by ethanol, evidenced by prominent differential splicing as well as transcript level-specific changes linked to altered excitatory neurotransmission.

### Paradigm-dependent transcriptomic effects of ethanol vary across brain regions

To investigate how acute and chronic ethanol exposure differentially influences gene expression in the brain, we performed paradigm × treatment interaction analysis for each region. Significant interaction genes primarily fell into two categories, both of which exhibited opposite expression effects between paradigms in response to ethanol: “Group A” genes were generally downregulated by acute but upregulated by chronic ethanol exposure, while “Group B” genes tended to be upregulated by acute but downregulated by chronic ethanol exposure (Fig. 6*A*; interaction analysis results in Extended Data Fig. 3-1*A*). In most brain regions, paradigm-dependent gene expression effects of ethanol were limited: There were two interaction genes in the dHPC, three in DS, four in VS, and 21 in cortex (Fig. 6*A*). Strikingly, however, there were 417 significant interaction genes in the vHPC (Fig. 6*A*). In the vHPC, the 136 Group A genes were significantly enriched for protein folding and IRE1-mediated unfolded protein response, while the 281 Group B genes were significantly enriched for biological processes such as iGluR signaling pathway, long-term synaptic depression, regulation of AMPA receptor activity, and downregulation of neuron apoptosis (Fig. 6*B*, Extended Data Fig. 6-1). Once again emphasizing regional specificity, nearly all of the 446 total significant interaction genes were specific to one brain region (Extended Data Fig. 6-2*A*). In fact, only one gene showed a significant interaction in more than one region: *Cacna1e* (calcium channel, voltage-dependent, R type, alpha 1E subunit) was identified as a Group B interaction gene in both the cortex and vHPC.

10.1523/ENEURO.0484-25.2026.f6-1 Figure 6-1 **Gene ontology enrichment for interaction genes in the ventral hippocampus.** Results from GO enrichment analysis for genes with significant paradigm × treatment interaction in the vHPC. GO enrichment results are reported in a direction-specific manner (i.e., for "upregulated" [Group A] or "downregulated" [Group B] DEGs). For Group B interaction genes, redundancy was reduced (similarity threshold=0.7) due to significant enrichment of >10 biological processes. Supports ***Figure 6B***. Download Figure 6-1, XLS file.

10.1523/ENEURO.0484-25.2026.f6-2 Figure 6-2 **Ethanol-induced gene and transcript expression signatures are region-specific. (A)** Upset plot showing overlap of genes with a significant paradigm × treatment interaction or similar ethanol-induced differential expression (DE) for both paradigms across brain regions. **(B)** Scatterplots of differential transcript expression for ethanol vs. saline within each paradigm, highlighting transcripts with significant paradigm × treatment interaction or similar ethanol-induced effects in both paradigms. **(C)** Upset plot showing overlap of transcripts with a significant paradigm × treatment interaction or similar ethanol-induced DE for both paradigms across brain regions. n=3-4 mice per group. Supports ***Figure 6***. See gene level differential expression results in ***Extended Data Figure 3-1*** and transcript level differential expression results in ***Extended Data Figure 5-1***. Download Figure 6-2, TIF file.

Across all regions, acute and chronic ethanol exposure induced similar expression changes in 201 genes, with 23 in the cortex (5 upregulated and 18 downregulated), 29 in dHPC (19 upregulated and 10 downregulated), 2 in vHPC (both *Calb1* [calbindin 1] and *Gfra1* [glial cell line derived neurotrophic factor family receptor alpha 1] upregulated), 117 in DS (36 upregulated and 81 downregulated), and 55 in VS (6 upregulated and 49 downregulated; Fig. 6*A*). Most of these were specific to one brain region (177 genes, ∼88%), but there were 24 genes with similar ethanol-induced DE between paradigms in more than one region (Extended Data Fig. 6-2*A*). For example, ethanol exposure significantly upregulated *Fkbp5* in the cortex, dHPC, and VS for both paradigms. Meanwhile, *Bahcc1* (BAH domain and coiled-coil containing 1) was significantly downregulated in the cortex and dHPC, while *Sh3bp5* (SH3-domain binding protein 5, BTK-associated) was upregulated in these regions. Interestingly, *Sh3bp5* was also a Group B interaction gene in the vHPC.

Transcript level paradigm × treatment interaction analysis revealed that the effects of ethanol were dependent on the extent of exposure for 1,542 transcript isoforms in the vHPC, 350 in cortex, 116 in VS, 8 in DS, and none in dHPC (Extended Data Fig. 6-2*B*; interaction analysis results in Extended Data Fig. 5-1). Similar to our gene level findings, we found the vast majority of interaction transcripts were specific to brain region (Extended Data Fig. 6-2*C*). In fact, only seven of the 2,009 interaction transcripts were not region specific: Three *Cacna1e* isoforms and two *Robo3* (roundabout guidance receptor 3) isoforms were Group B interaction transcripts in the cortex and vHPC, while two *Xlr3b* (X-linked lymphocyte-regulated 3B) isoforms were Group A interaction transcripts in the cortex and VS. Interestingly, additional transcript isoforms of these genes were identified as interaction transcripts in only one region: Two *Cacna1e* isoforms were Group B interaction transcripts in the vHPC, two *Robo3* isoforms were Group B transcripts in cortex, and one *Xlr3b* isoform was a Group A transcript in cortex. These findings further indicate ethanol-induced transcript expression changes are predominantly specific to brain region.

As at the gene level, acute and chronic ethanol exposure induced similar transcript expression changes the most in the DS (301 DETs), which was subsequently followed by 180 in the VS, 74 in dHPC, 59 in cortex, and 8 in vHPC (Extended Data Fig. 6-2*B,C*). Across all regions, acute and chronic ethanol exposure induced similar expression changes in 562 transcripts, most of which were specific to one region (507 transcripts, ∼90%; Extended Data Fig. 6-2*C*).

Next, we examined the extent of genes showing significant interaction at both the gene and transcript levels. In the vHPC, nearly two thirds of interaction transcripts were encoded by interaction genes (336 genes), while over one third of interaction transcripts were not (195 interaction TO-genes; Fig. 6*C*). In stark contrast, interaction transcripts in the cortex, DS, and VS were primarily encoded by interaction TO-genes rather than by interaction genes (Fig. 6*C*).

Finally, we compared genes differentially spliced in response to ethanol between acute and chronic paradigms. For each region, genes with ethanol-induced AS were predominantly specific to the extent of exposure (≥87% of all DSGs per region; Fig. 6*D*; splicing analysis results in Extended Data Fig. 5-4), suggesting that the extent of ethanol exposure largely influences which genes are differentially spliced. Interestingly, the fractions of paradigm-specific DSGs were nearly even in each region except the DS, where paradigm-specific DSGs were strongly biased toward acute exposure (∼72% of DSGs in the DS; Fig. 6*D*).

Taken together, these findings indicate different brain regions show differing levels of paradigm-specific gene and transcript expression changes in response to ethanol, and DSGs largely differ between acute and chronic ethanol exposure paradigms. In addition, the vHPC emerged as a brain region with ethanol-induced expression changes that are overwhelmingly dependent on the extent of exposure, suggesting transcriptomic changes in the vHPC might play a relevant role in the transition from limited to chronic alcohol consumption.

## Discussion

Here, we explored the epigenetic and transcriptomic effects of acute and chronic ethanol exposure, focusing on key brain regions relevant to AUD (Koob and Volkow, 2016). We showed ethanol-derived acetate is incorporated into histone acetylation in the brain following acute or chronic exposure, and this was more robust following repeated exposure. Further, we found chromatin and transcriptomic changes elicited by acute or chronic ethanol exposure are predominantly specific to brain region. Interestingly, ethanol-induced molecular changes were paradigm dependent in some but not all brain regions, and we noted particularly profound paradigm-dependent transcriptomic effects of ethanol in the vHPC. Overall, this study systematically illuminates critical epigenetic and transcriptomic outcomes linked to ethanol exposure and how these molecular outcomes differ between acute and chronic exposure, thus providing valuable insight for developing novel therapeutics for AUD.

Our heavy labeling findings corroborated previous reporting of ethanol-derived acetate being directly incorporated into histone acetylation in the mouse HPC following acute exposure (Mews et al., 2019). Here, we expanded upon this work by investigating additional brain regions linked to AUD in the context of not only acute but also chronic exposure. Ethanol-derived acetate from a single exposure contributed to histone acetylation in the HPC and VS but not cortex or DS (Fig. 1*B*), whereas ethanol-derived acetate from chronic exposure contributed to histone acetylation in each region (Fig. 1*C*). Our findings suggest different brain regions have differing levels of sensitivity to this metabolic-epigenetic axis, and this sensitivity may vary depending on extent of exposure. However, the underlying mechanisms remain unclear and warrant further investigation. For example, region-specific molecular outcomes could be attributed to differences in the abundance of specific cell types among brain regions, which could confound proteomic and transcriptomic results from tissue homogenates. Similarly, the spatiotemporal distribution of alcohol and alcohol-derived acetate throughout the brain following acute and chronic treatment, as well as the expression and subcellular localization of ACSS2 in various brain regions both at baseline and following alcohol exposure, will need to be further investigated to determine their potential contributions to the observed epigenetic and transcriptomic outcomes.

Importantly, acetylation and methylation changes occurred at similar frequencies, indicating the epigenetic effects of ethanol extend beyond direct incorporation of ethanol-derived acetate into histone acetylation. H2A.Z.1K11ac was the only hPTM affected in the same region in both paradigms, showing increased hippocampal abundance following acute or chronic ethanol exposure (Fig. 2*A*,*B*), consistent with a significant main effect of treatment (Fig. 2*C*). Notably, acetylated H2A.Z mediates transcriptional outcomes that contribute to learning and memory (Narkaj et al., 2018; Reda et al., 2024; Periandri et al., 2026).

The epigenetic effects of ethanol were largely region-specific. All hPTMs significantly altered by acute or chronic ethanol were restricted to one region, with H3K36me3 as the exception: H3K36me3 was reduced in the cortex following acute (Fig. 2*A*) but increased in the DS and VS following chronic (Fig. 2*B*) ethanol exposure, demonstrating divergent regional effects between paradigms. H3K36me3 is linked to AS in the brain (Hu et al., 2017, 2020) and has been implicated in substance-related behaviors. For example, H3K36me3-mediated AS regulates cocaine reward-related behavior (Xu et al., 2021), and adolescent binge ethanol exposure disrupts cortical H3K36me3 enrichment at synaptic genes such as *Cacna1a* (calcium channel, voltage-dependent, P/Q type, alpha 1A subunit; Brocato and Wolstenholme, 2023). Interestingly, we observed reduced cortical H3K36me3 abundance alongside downregulated expression of *Cacna1a* and of six of its 13 transcript isoforms following acute ethanol exposure.

Ethanol produces molecular, synaptic, and circuit level adaptations varying across brain regions (Abrahao et al., 2017), and alcohol's lasting transcriptomic effects are distinct across brain regions (Contet, 2012; Warden and Mayfield, 2017; Van Booven et al., 2021; Huggett et al., 2023; Willis et al., 2025). Our findings strongly support this: Ethanol-induced changes to gene and transcript expression and AS (Figs. 3–5) showed stark regional specificity. In fact, only two genes were similarly affected across all regions: *Fkbp5* and *Mt2* were upregulated in each region following acute ethanol exposure (Fig. 3*D*). Interestingly, both of these genes have been previously linked to alcohol. Single-nucleotide polymorphisms in human *FKBP5* have been associated with alcohol withdrawal severity and alcohol drinking, and *Fkbp5* knock-out in mice heightens alcohol withdrawal severity and alcohol consumption (Huang et al., 2014; Qiu et al., 2016). Voluntary drinking increases astrocytic *Mt2* expression in mice (Mulligan et al., 2011), and MT family genes are upregulated in various brain regions of postmortem AUD patients (Friske et al., 2025).

Gene and transcript expression changes were more extensive after acute than chronic ethanol exposure (Figs. 3–5). Similar patterns were observed in BXD mice (van der Vaart et al., 2017), yet systematic transcriptomic comparisons between acute and chronic exposure remain limited. The pronounced transcriptional response to acute exposure largely comprises transient changes not persisting with chronic exposure, possibly reflecting allostatic adaptations to repeated exposure such as desensitization, a neuroadaptation underlying tolerance (Dopico and Lovinger, 2009; Bhandari et al., 2024). Notably, acute ethanol exposure induced transient expression changes to RGs, and this dysregulation was muted after chronic exposure (Extended Data Fig. 3-3). RGs regulate synaptic plasticity (recently reviewed in Khan et al., 2025) and have been linked to alcohol (Pignataro et al., 2007; Larnerd et al., 2023).

Aberrant AS in the brain strongly correlates with chronic alcohol consumption (Van Booven et al., 2021; Huggett et al., 2023; Carvalho and Lasek, 2024; Bhatnagar and Heller, 2025), which is supported by our current findings (Fig. 5*I*). Surprisingly, however, few studies have investigated AS in the context of acute ethanol exposure (Signor and Nuzhdin, 2018; Wolfe et al., 2019). Here, we revealed hundreds of ASEs in AUD-related brain regions after acute ethanol exposure (Fig. 5*D*,*E*). The number of DSGs was comparable between paradigms, while the specific DSGs largely differed (Fig. 6*D*), underscoring the importance of investigating differential AS in the context of acute exposure.

DSGs in the brain rarely overlap with DEGs (Stilling et al., 2014; Xu et al., 2021; Bhatnagar et al., 2023; Bhatnagar and Heller, 2025). Indeed, most DSGs did not correspond to DEGs or DETs (Fig. 5*F*,*L*). For example, *Stat3* did not differ in expression but was a DSG: Chronic ethanol exposure significantly decreased inclusion of an A3SS event of *Stat3* exon 23 in the vHPC (Extended Data Fig. 5-2*C*). Interestingly, AS of human *STAT3* exon 23 promotes the switch from the full-length STAT3α isoform to the dominant negative STAT3β isoform that is truncated and lacks a transactivation domain (Wang et al., 2019). STAT has also been implicated in alcohol-related outcomes. In fruit flies, *Stat92E* is differentially spliced after alcohol memory formation (Petruccelli et al., 2018, 2020) or repeated ethanol exposure (Wilson et al., 2023) and is required in memory-encoding mushroom body neurons for lasting alcohol-associative preference (Petruccelli et al., 2018, 2020) and suppressing ethanol sensitization (Wilson et al., 2023). In rodents, *Stat3* regulates hippocampal gene expression during alcohol withdrawal (Chen et al., 2021), and STAT3 inhibition reduces binge-like ethanol drinking (Hamada et al., 2021).

The vHPC is particularly sensitive to ethanol. Chronic ethanol consumption results in greater pyramidal neuron loss in the vHPC than dHPC (Lescaudron and Verna, 1985), and chronic intermittent ethanol exposure increases synaptic excitability in the vHPC but not dHPC (Ewin et al., 2019). Chronic low-dose ethanol exposure alters vHPC regulation of reward seeking (Bryant et al., 2023), and the vHPC negatively regulates alcohol drinking (Griffin et al., 2023). Here, we found ethanol-induced expression changes in vHPC were strikingly dependent on paradigm (Fig. 6*A*, Extended Data Fig. 6-1*B*). Group A interaction genes were enriched for proteostasis-related processes, including unfolded protein response (UPR; Fig. 6*B*). The UPR is activated in the pancreas as an adaptive response to chronic alcohol-induced ER stress (Lugea et al., 2011), raising the possibility of a similar adaptive response in the vHPC. Further, UPR signaling supports synaptic plasticity and memory formation (Martínez et al., 2018) but can also contribute to neurodegeneration (Duran-Aniotz et al., 2017), thus the observed opposing effects between paradigms may reflect shifts between protective and maladaptive states. In contrast, Group B interaction genes were primarily enriched for neurotransmission-related processes (Fig. 6*B*). Transient upregulation of genes involved in synaptic plasticity suggests acute enhancement of synaptic remodeling, whereas their chronic downregulation suggests reduced plasticity with repeated exposure. Likewise, acute upregulation of genes linked to excitatory neurotransmission indicates a short-term increase in vHPC excitability, while the opposing chronic downregulation may reflect the dampening of excitatory signaling that characterizes neuroadaptations underlying tolerance.

Consistent with interaction patterns, downregulated DEGs in the vHPC following chronic ethanol exposure related to glutamatergic synaptic transmission (Fig. 4*B*) and TO-genes mapped to iGluR signaling (Fig. 5*H*). The vHPC provides major glutamatergic input to the nucleus accumbens (NAc; Britt et al., 2012), a key component of the VS reward circuitry. Chronic ethanol exposure enhances glutamatergic vHPC → NAc input (Kircher et al., 2019), and alcohol dependence disrupts the inhibitory function these projections exert on alcohol consumption (Griffin et al., 2023). Thus, the ventral hippocampal downregulation of genes involved in glutamatergic signaling may reflect a compensatory response to elevated excitatory circuit activity.

Future work is critical to address important limitations of the current study. Here, we delivered a precise dose to each mouse via intraperitoneal injections, enabling the reliable detection of stable isotope incorporation into brain histone acetylation. This controlled approach provides reproducible ethanol dosing across animals, reducing interindividual variability and enabling clearer inference between ethanol exposure and molecular outcomes. However, intraperitoneal injections may subject animals to stress and do not recapitulate human voluntary consumption behaviors. Future studies will utilize alternative models of exposure that better approximate human alcohol intake (e.g., voluntary drinking or gavage), which will also allow us to evaluate how molecular profiles correlate with relevant alcohol-related behaviors (e.g., preference, craving/seeking, binge drinking). Another notable limitation here is the exclusive use of male mice, rationalized by the higher prevalence of AUD diagnosis in males (World Health Organization, 2024). However, this gap between sexes is converging (McKee and McRae-Clark, 2022), and there is accumulating evidence for sex-dependent transcriptional responses to alcohol (Hitzemann et al., 2022) and sex-dependent epigenetic, transcriptional, and behavioral responses to the alcohol metabolite acetate (Periandri et al., 2026) in the brain. In addition, emerging evidence points to sex differences in efficacy and adverse effects of pharmacotherapeutic interventions for substance use disorders (McKee and McRae-Clark, 2022), further necessitating the characterization of sex differences in the brain's epigenetic and transcriptomic responses to alcohol.

Here, we provide systematic new insights into epigenetic, gene and transcript expression, and splicing changes across key AUD-related brain regions following acute or chronic ethanol exposure, underlining distinct regional specificity. Our study is one of few exploring how ethanol-induced transcriptomic outcomes differ by extent of exposure and identified a particularly striking effect in the vHPC. Collectively, these findings and follow-up work systematically addressing cell-type specificity and sex differences are critical for understanding epigenetic and transcriptomic responses to ethanol, ultimately informing novel therapeutics for AUD.

## Synthesis

Reviewing Editor: Sam Golden, The University of Washington Decisions are customarily a result of the Reviewing Editor and the peer reviewers coming together and discussing their recommendations until a consensus is reached. When revisions are invited, a fact-based synthesis statement explaining their decision and outlining what is needed to prepare a revision will be listed below. The following reviewer(s) agreed to reveal their identity: Elizabeth Heller-Mesznik. Note: If this manuscript was transferred from JNeurosci and a decision was made to accept the manuscript without peer review, a synthesis may not be available.

Dear Dr. Egervari,

Your manuscript has been reviewed by two experts in the field, who both found the manuscript of interest and technical merit. Both reviewers provided relatively minor comments that must be addressed, which will improve the final manuscript. I look forward to reviewing your rebuttal and revised manuscript. Below please find the reviewer comments:

Reviewer 1:

Periandri et al present a comprehensive profiling of acute and chronic alcohol exposure-induced changes in chromatin and gene expression across several brain regions. The work is novel in its study of direct incorporation of alcohol metabolized acetyl groups into histones in these brain regions, using innovative heavy-label isotope mass spec profiling across brain regions and treatment durations. The study complements a recent manuscript by the group which found that knockout of ACSS2, the enzyme necessary for histone acetylation downstream of alcohol, is also necessary for male alcohol drinking, gene expression changes, and histone acetylation in the ventral striatum. This prior study is furthered in the current manuscript by expanding the effects of alcohol on histones and gene expression to multiple brain regions. The current manuscript also nicely reproduced hpc findings from the prior study, serving as a biological replicate and positive control. The study is rigorous with appropriate controls, the methods are described in depth, and the conclusions are supported by the data. The minor comments below are intended to improve clarity.

1. Although not directly analyzed here, the authors prior study elucidated gene expression changes following drinking-in-the-dark. How do DEGs compare between that treatment paradigm and the current investigator-administered paradigm?

2. Please revise to justify the lack of female subjects.

3. What is the rationale and method for performing transcript vs gene level differential expression analyses? These largely overlap, which is encouraging. Why would the DETs and DEGs differ?

4. Please report the read depth >50M reads - sufficient for rMATS splicing analysis.

Reviewer 2:

This is a well-written paper examining the effects of acute and chronic IP ethanol administration in male mice on the brain transcriptome and epigenetic modifications. Mice were administered heavy-labeled 20% ethanol to measure histone acetylation from ethanol-derived acetate and histone peptide modifications using LC-MS/MS. In a separate group of mice, RNA was extracted and subjected to next-generation sequencing. The authors compared the effects of ethanol on epigenetic modifications and the transcriptome in the hippocampus, dorsal striatum, ventral striatum and cortex. Comparing various brain regions and effects of acute vs. chronic ethanol exposure are powerful approaches that add important knowledge of ethanol's effects on gene expression in the brain. My main comments concern discussing some of the limitations of the study and clarifying a few details in the manuscript.

1. The animal species used for these studies should be indicated in the abstract.

2. The source of the C57BL/6J mice should be indicated in the animals and experimental design section (i.e., purchased from JAX or bred in-house).

3. The number of mice used for each experiment should be indicated in the animals and experimental design section.

4. The authors should indicate if there is any potential confound of using different library preparation kits and sequencing platforms when comparing differential gene expression between brain regions.

5. The authors should briefly discuss the limitations (and advantages) of the approach of using IP injections for chronic alcohol exposure and studying effects of ethanol in only male mice.

6. Data should be deposited into NCBI Gene Expression Omnibus (GEO) and the accession number provided prior to acceptance of this paper. The data does not need to be made public in GEO until the date of publication.

7. For the isotope labeling/mass spectrometry, please indicate whether the whole hippocampus was analyzed or a subregion.

8. One reason for region-specific effects on DEGs in response to ethanol exposure could be due to the abundance of different cell types in these brain regions. This should be acknowledged in the discussion.

9. The authors describe results of Stat3 alternative splicing in the discussion but the results are not shown in a figure. These results are very interesting. Adding this specific example to a figure would increase the impact of the study.

10. What are potential reasons for differences between ethanol-derived acetate incorporation between brain regions?
