# Supplementary figures and images for: Epigenetic and Transcriptomic Impacts of Ethanol Vary by Brain Region and Extent of Exposure
Source: eNeuro. 2026 Apr 9;13(4):ENEURO.0484-25.2026. doi: 10.1523/ENEURO.0484-25.2026 (PMC13086551; doi:10.1523/ENEURO.0484-25.2026)

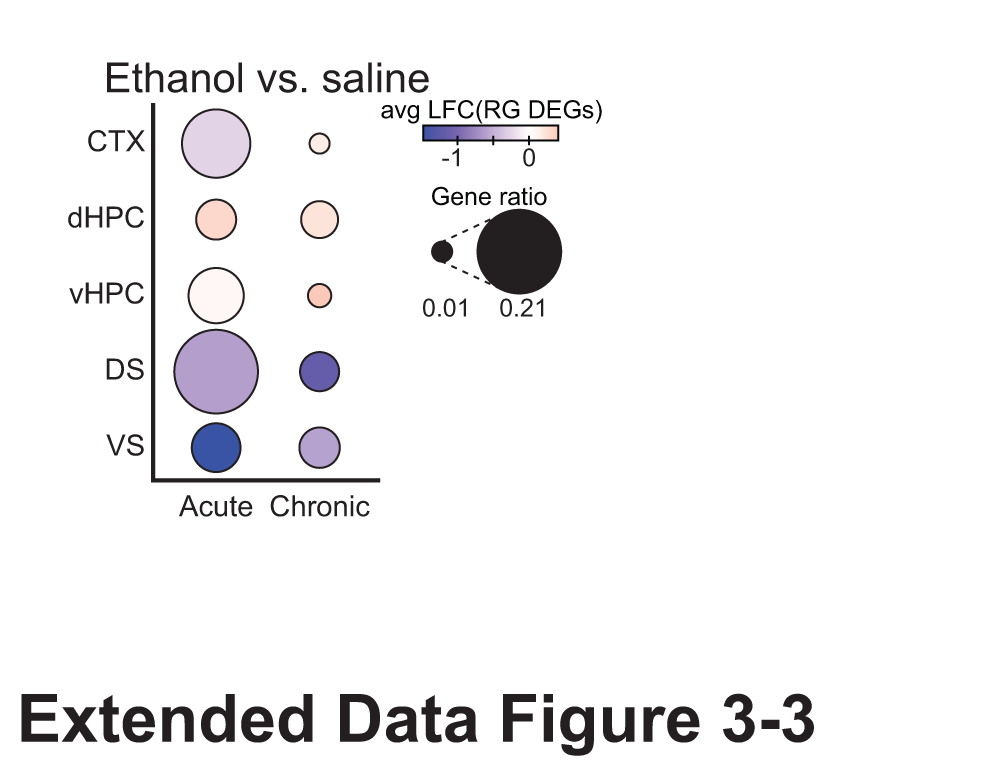

Supplement: Figure 3-3 — Differential expression of response genes in response to ethanol treatment. Dot plot of response gene (RG) DEGs for each region following acute or chronic ethanol exposure. n=3-4 mice per group. See Extended Data Figure 3-1 for gene level differential expression results and queried RGs. Download Figure 3-3, TIF file. [file eneuro-13-ENEURO.0484-25.2026-s005.tif]

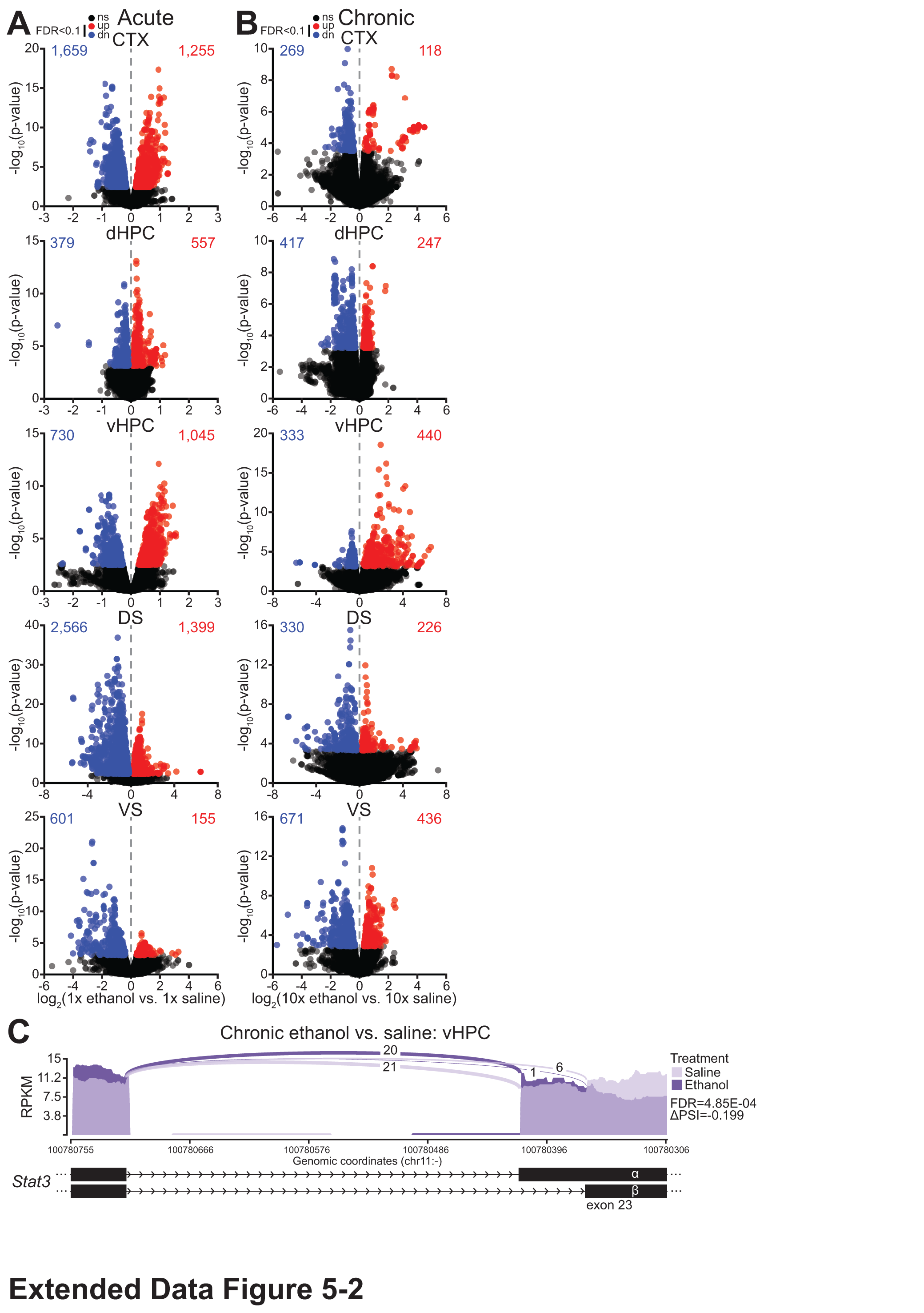

Supplement: Figure 5-2 — Transcript isoform expression and alternative splicing are dysregulated in the brain following ethanol treatment. (A-B) Volcano plots of differential transcript expression in each brain region in response to (A) acute ethanol exposure and (B) chronic ethanol exposure. (C) Sashimi plot generated using rmats2sashimiplot showing a significant alternative 3' splice site (A3SS) event of Stat3 exon 23 in the vHPC in response to chronic ethanol exposure. Genomic coordinates span Stat3 exon 23 and the alternative 3' splice junctions corresponding to the full-length (α) and truncated (β) forms. Normalized read coverage (RPKM) is shown for each treatment group. Curved arcs represent splice junctions, with overlaid numbers denoting the average number of reads supporting the junction per sample within the group. n=3-4 mice per group. Abbreviations: ΔPSI, change in percent spliced in; RPKM, reads per kilobase million. Supports Figure 5. See Extended Data Figure 5-1 for transcript level differential expression results and Extended Data Figure 5-4 for alternative splicing results. Download Figure 5-2, TIF file. [file eneuro-13-ENEURO.0484-25.2026-s008.tif]

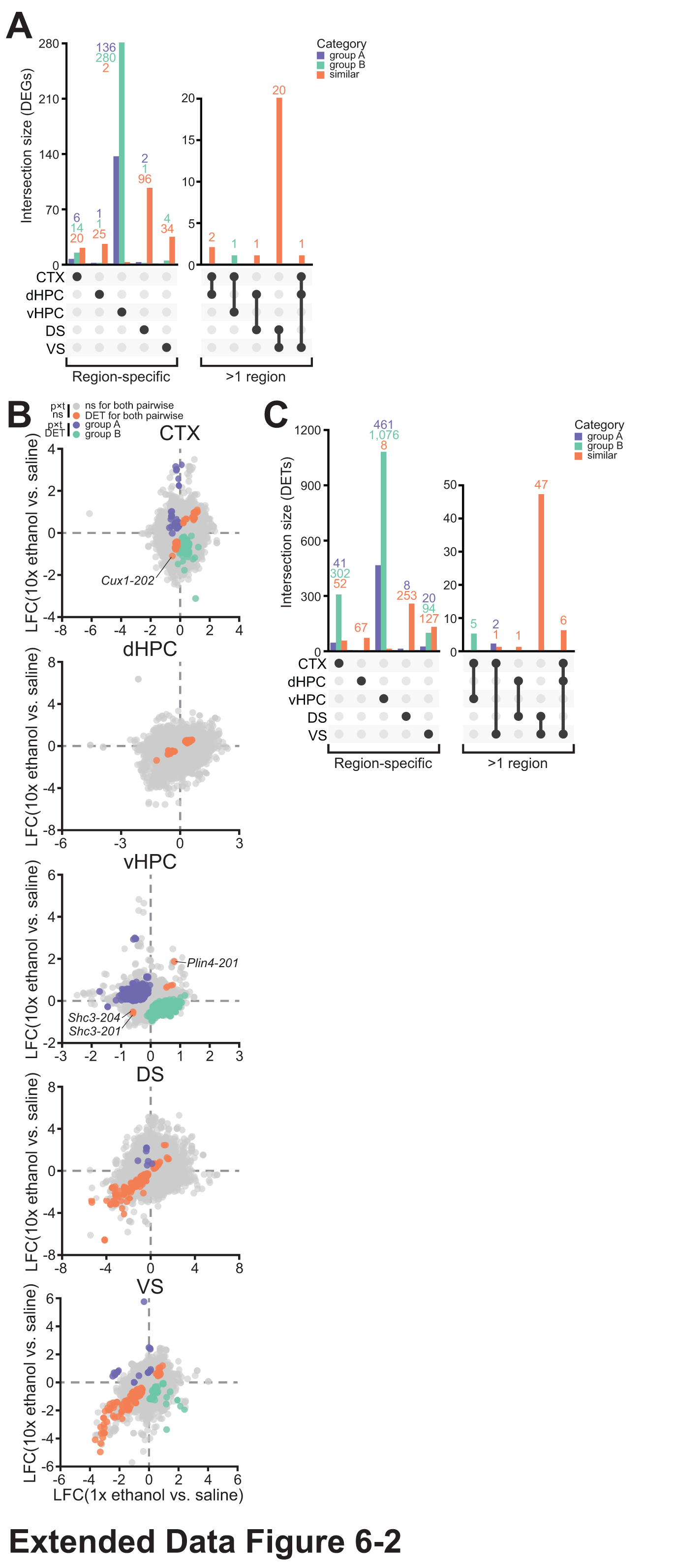

Supplement: Figure 6-2 — Ethanol-induced gene and transcript expression signatures are region-specific. (A) Upset plot showing overlap of genes with a significant paradigm × treatment interaction or similar ethanol-induced differential expression (DE) for both paradigms across brain regions. (B) Scatterplots of differential transcript expression for ethanol vs. saline within each paradigm, highlighting transcripts with significant paradigm × treatment interaction or similar ethanol-induced effects in both paradigms. (C) Upset plot showing overlap of transcripts with a significant paradigm × treatment interaction or similar ethanol-induced DE for both paradigms across brain regions. n=3-4 mice per group. Supports Figure 6. See gene level differential expression results in Extended Data Figure 3-1 and transcript level differential expression results in Extended Data Figure 5-1. Download Figure 6-2, TIF file. [file eneuro-13-ENEURO.0484-25.2026-s012.tif]
